# Serum levels of apolipoprotein A-I and high-density lipoprotein can predict organ failure in acute pancreatitis

**DOI:** 10.1186/s13054-015-0832-x

**Published:** 2015-03-17

**Authors:** Yun-Shing Peng, Yung-Chang Chen, Ya-Chung Tian, Chih-Wei Yang, Jau-Min Lien, Ji-Tseng Fang, Cheng-Shyong Wu, Chien-Fu Hung, Tsan-Long Hwang, Ying-Huang Tsai, Mel S Lee, Ming-Hung Tsai

**Affiliations:** Division of Endocrinology and Metabolism, Chang Gung Memorial Hospital, 6, West Section, Chia-Pu Road, Chia-Yi, Taiwan; Division of Critical Care Nephrology, Chang Gung Memorial Hospital, 199, Tung-Hwa North Road, Taipei, Taiwan; Division of Digestive Therapeutic Endoscopy, Chang Gung Memorial Hospital, 199, Tung-Hwa North Road, Taipei, Taiwan; Division of Gastroenterology, Chang Gung Memorial Hospital, 6, West Section, Chia-Pu Road, Chia-Yi, Taiwan; Department of Radiology, Chang Gung Memorial Hospital, 199, Tung-Hwa North Road, Taipei, Taiwan; Division of General Surgery, Chang Gung Memorial Hospital, 199, Tung-Hwa North Road, Taipei, Taiwan; Division of Thoracic and Critical Care Medicine, Chang Gung Memorial Hospital, 6, West Section, Chia-Pu Road, Chia-Yi, Taiwan; Department of Surgery, Chang Gung Memorial Hospital, 6, West Section, Chia-Pu Road, Chia-Yi, Taiwan; Chang Gung University, College of Medicine, 259, Wen-Hwa 1st Road, Kwei-Shan, Tao-Yuan, Taiwan; Division of Gastroenterology and Hepatology, Chang Gung Memorial Hospital, Chang Gung University, 199, Tung-Hwa North Road, Taipei, 105 Taiwan

## Abstract

**Introduction:**

Predicting severity of pancreatitis is an important goal. Clinicians are still searching for novel and simple biomarkers that can better predict persistent organ failure (OF). Lipoproteins, especially high-density lipoprotein (HDL), and apolipoprotein A-I (APO A-I), have been shown to have anti-inflammation effects in various clinical settings. Severe acute pancreatitis (SAP) is associated with hypo-lipoproteinemia. We studied whether the concentrations of HDL and APO A-I can predict persistent OF in patients with predicted SAP admitted to the ICU.

**Methods:**

In 66 patients with predicted SAP, we prospectively evaluated the relationship between lipid levels, inflammatory cytokines and clinical outcomes, including persistent OF and hospital mortality. Blood samples were obtained within 24 hours of admission to the ICU.

**Results:**

HDL and APO A-I levels were inversely correlated with various disease severity scores. Patients with persistent OF had lower levels of HDL and APO A-I, while those with transient OF had lower levels of interleukin-6, tumor necrosis factor-α and lower rates of hospital mortality. Meanwhile, hospital non-survivors had lower concentrations of HDL, and APO A-I compared to the survivors. By using the area under the receiver operating characteristic (AUROC) curve, both HDL and APO A-I demonstrated an excellent discriminative power for predicting persistent OF among all patients (AUROC 0.912 and 0.898 respectively) and among those with OF (AUROC 0.904 and 0.895 respectively). Pair-wise comparison of AUROC showed that both HDL and APO A-I had better discriminative power than C-reactive protein to predict persistent OF.

**Conclusions:**

Serum levels of HDL and APO A-I at admission to the ICU are inversely correlated with disease severity in patients with predicted SAP and can predict persistent OF in this clinical setting.

**Electronic supplementary material:**

The online version of this article (doi:10.1186/s13054-015-0832-x) contains supplementary material, which is available to authorized users.

## Introduction

Acute pancreatitis represents an acute inflammatory disorder with variable severity ranging from mild, self-limited disease to a severe inflammatory cascade associated with multiple-organ failure. Despite the improvements in critical care, severe acute pancreatitis (SAP) is still associated with a high mortality rate [1]. Although the incidence of organ failure in the early phase (within the first week) varies among different studies [2-6], persistent organ failure (OF) has been shown to be the major determinant of clinical outcomes [6,7]. These observations have led to modified definitions of SAP, which recognize the importance of persistent OF and introduce a new sub-classification of moderately severe pancreatitis based on the presence of transient OF [8,9].

It is important to stratify the severity of acute pancreatitis. First, early identification of patients with potential SAP may facilitate timely referral to specialists. Second, for specialists, severity-stratification of those patients makes triage and comparison between different studies possible. However, when organ failure is present in the early phase, it may be difficult to determine the final degree of severity because it is unknown whether the patient will prove to have persistent or transient OF.

It has been shown that the concentrations of total cholesterol and lipoproteins decrease in the early stages of critical illness [10-12]. Indeed, the pattern of early rapid decline is found primarily in the high-density lipoprotein (HDL) [12]. Moreover, the protein composition of HDL is markedly altered as well, with the apolipoprotein A1 (APO A-I) depleted [12].

Apo A-I is the major protein component of HDL. APO A-I plays an essential role in cholesterol homeostasis by transporting excessive cholesterol from extra-hepatic tissues to the liver. Aside from this biological function, recently, more emphasis has been placed upon its anti-inflammatory effects. Apo A-I can attenuate lipopolysaccharide-induced cytokine secretion in a mode of protein-endotoxin interaction, even without its physiological lipid complement [13,14]. Furthermore, elevated cytokines can downregulate hepatic synthesis of HDL and APO A-I [15].

Although there have been studies of lipoprotein profile in acute pancreatitis [16-18], there has been no report on the specific role of lipoprotein in predicting severity using the most updated definition. The results of previous studies raise the possibility that the levels of lipoprotein may play a role in assessing disease severity [17,18]. However, these studies had several limitations, including retrospective design [17], and inconsistent definitions of severity ranging from severe pancreatitis based on the Atlanta criteria to mortality [17,18]. None of these studies evaluated persistent OF, which has become recognized as the most clinically relevant indicator of disease severity. Furthermore, these studies have mainly focused on the analysis of total cholesterol, triglycerides, LDL or HDL cholesterol. APO A-I levels have rarely been reported, even though APO A-I represents the major protein component of HDL, modulating inflammation.

We hypothesized that low levels of lipoprotein are associated with a more pronounced inflammatory state in patients with predicted SAP and may serve as a marker to discriminate persistent OF from transient OF upon admission to ICU. Therefore, we conducted this prospective observational investigation to study whether low concentrations of APO A-I and HDL are associated with persistent OF and hospital mortality. Other potential indicators of inflammation such as C-reactive protein (CRP), TNF-α and IL-6 were also measured.

## Material and methods

### Patient information, data collection and definitions

This study was conducted with the approval of the institutional review board of Chang Gung Memorial Hospital, Taiwan and in accordance with the Declaration of Helsinki of the World Medical Association. Formal consent was obtained from the patient or the next of kin. The study was performed in the ICU of two university-affiliated hospitals between February 2008 and September 2011. This study enrolled 66 patients with predicted SAP requiring intensive monitoring and/or treatment. Predicted SAP was defined by acute physiology, age, and chronic health evaluation (APACHE II) score ≥8 at admission [19]. All patients included in this study were admitted to hospital within 72 hours of onset of symptoms.

The diagnosis of acute pancreatitis was made based on two or more of the following: typical abdominal pain; serum amylase and/or lipase levels at least three times the upper limit of normal; and characteristic findings of acute pancreatitis on a contrast-enhanced computed tomography (CT) scan, or abdominal ultrasonography. Abdominal ultrasonography was performed for each case at presentation. Enhanced CT was performed when the disease was evaluated as predicted SAP. CT-guided aspiration was performed and bacterial cultures were obtained when infected necrosis was suspected.

Organ failure was defined as a score of ≥2 in one or more of the three (respiratory, renal, and cardiovascular) organ systems described in the modified Marshall score during the first week [20,21]. This definition is in accordance with the most updated revised Atlanta classification [8,9]. OF was defined as transient for duration <48 hours or persistent if >48 hours. Meanwhile, disease severity also was assessed by sequential organ failure assessment (SOFA) [22].

Patients with a history of prior acute pancreatitis or drug history of lipid-lowering agents, and patients those receiving total parenteral nutrition at the time of blood sampling were excluded from this study. All ICU admissions were followed until discharge from the hospital or hospital mortality.

### Laboratory investigations

Blood cultures and appropriate cultures from the infection focus were obtained. Hematological and biochemical data including serum lipoprotein were also collected systemically within 24 hours of admission to ICU, with a median of 3 days (IQR 2 to 4 days) after the start of symptoms and a median of 2 days (IQR 1 to 3 days) after admission to hospital. For the scoring systems, the most abnormal value for each organ system on the day of blood sampling was recorded.

A fasting blood sample was obtained in the morning. The blood samples were allowed to clot and were spun immediately in a refrigerated centrifuge. The serum was obtained and frozen at −80°C. Total cholesterol (TC) and triglyceride (TG) were measured enzymatically using kits (Wako Pure Chemical, Osaka, Japan). The concentrations of HDL and LDL were directly measured using a homogenous assay (Daiichi Pure Chemical, Ibaraki, Japan). APO A-I was measured by means of immunoturbidimetry with the TurbiTime system (Dade Behring Inc, Newark, DE, USA). The concentrations of TNF-α and IL-6 were measured by an enzyme-linked immunosorbent assay (R & D Systems, Minneapolis, MN, USA). CRP was measured by a latex-enhanced immunoturbidimetric method (Daiichi Pure Chemical).

### Statistical analysis

Descriptive statistics are expressed as mean ± SD. All variables were tested for normal distribution using the Kolmogorov-Smirnov test. The Student *t*-test was used to compare the means of continuous variables and the normality of data distribution. Otherwise, the Mann-Whitney *U*-test was used. Categorical data were tested using the chi-square (*χ*^2^) test. The Kruskall-Wallis test followed by Dunn’s post hoc test was used for multiple comparison. Correlation between the lipid levels, cytokines and the disease severity scores was analyzed with the Spearman rank method. Discrimination was tested using the area under a receiver operating characteristic (ROC) curve (AUROC) to assess the ability of HDL cholesterol and APO A-I to predict persistent OF. ROC analysis was also performed to calculate the cutoff values, sensitivity, specificity, overall correctness, and positive and negative predictive values (PPV and NPV). The best Youden index (sensitivity + specificity-1) [23] was also used to determine the best cutoff point of HDL and APO A-I to predict persistent OF. All statistical tests were two-tailed, and the significance level was set at *P* ≤0.05. Data were analyzed using SPSS 18.0 for Windows (SPSS Inc., Chicago, IL, USA) except for comparisons of ROC curve. The comparisons between ROC curves were calculated with the MedCalc software (MedCalc Software 14.12.0, Belgium), using the Hanley and McNeil method [24].

## Results

### Subject characteristics

During the study period, 69 consecutive patients with predicted SAP were admitted to ICU: 3 patients were excluded because of prior history of acute pancreatitis, and 66 patients were enrolled in this investigation. Table 1 lists the patients’ demographic data and clinical characteristics. Overall, the in-hospital mortality for the entire group was 19.7%. Compared to hospital survivors, the hospital non-survivors had higher disease severity as evidenced by higher Ranson, APACHE II, SOFA, and modified Marshall scores.

**Table 1:** **Patients’ demographic data and clinical characteristics**

	**Hospital survivors**	**Hospital non-survivors**	***P*** **-value**
	**(n = 53)**	**(n = 13)**	
Age, years	60 (45 to 76.5)	59 (45 to 84)	0.640
Gender, M/F, number	29/24	9/4	0.532
BMI, kg/m^2^	25.9 ± 4.0	25.2 ± 3.7	0.597
Body weight, kg	67.6 ± 12.1	69.2 ± 11.8	0.671
Etiology, alcohol/biliary/ERCP/others	19/31/0/3	6/6/1/0	0.145
Leukocytes, ×10^9^/l	11.8 ± 5.7	14.6 ± 0.9	0.301
Hemoglobin, g/dl	10.4 ± 2.1	10.3 ± 2.6	0.893
Platelets, ×10^9^/l	157.5 (88.3 to 241)	62 (25 to 216)	0.043
BUN, mg/dl	17.5 (10.8 to 26.5)	44 (14.5 to 91.5)	0.009
Serum creatinine, mg/dl	0.9 (0.7 to 1.9)	3.1 (1.8 to 5.4)	0.003
Bilirubin, mg/dl	1.5 (0.9 to 3)	8 (2.7 to 22. 5)	<0.001
INR	1.2 (1.1 to 1.4)	1.5 (1.2 to 1.8)	0.007
Ranson score	5 (4 to 6)	6 (6 to 8.5)	0.001
APACHE II score	15 (11 to 20.8)	28 (16.5 to 34.8)	0.002
SOFA score	5 (3 to 8)	13 (6.5 to 15.8)	0.001
Modified Marshall score	3 (2 to 5.5)	9.5 (7 to 10.8)	<0.001
Organ failure	42 (79.2%)	13 (100%)	0.104
Number of organ failures	1 (1 to 2)	3 (2 to 3)	<0.001
Respiratory failure	38 (71.7%)	12 (92.3%)	0.162
Renal failure	21 (39.6%)	11 (84.6%)	0.005
Cardiovascular failure	13 (24.5%)	11 (84.6%)	<0.001
Infected necrosis, n	10 (18.9%)	8 (61.5%)	0.002
Persistent organ failure, n	22 (41.5%)	13 (100%)	<0.001
Cholesterol, mg/dl	122 (92 to 152)	98 (81 to 139)	0.256
Triglyceride, mg/dl	140 (107 to 239)	180 (102 to 362)	0.723
HDL, mg/dl	17 (11 to 31)	6 (4 to 13)	<0.001
LDL, mg/dl	61 (40 to 79)	43 (30 to 91)	0.297
APO A-I, mg/dl	67 (50 to 83)	40 (23 to 46)	<0.001
IL-6, pg/ml	73 (39.7 to 177.8)	388 (125 to 831.5)	0.015
TNF-α, pg/ml	6.2 (2.9 to 13.5)	25 (20 to 42)	0.011
CRP, mg/l	182.6 ± 115.6	151.6 ± 77.4	0.382

Results are presented as mean ± SD, or median (IQR) unless otherwise stated. M, male; F, female; BMI, body mass index; ERCP, endoscopic retrograde cholangiopancreagraphy; BUN, blood urea nitrogen; INR, international normalized ratio; APACHE, acute physiology, age, and chronic health evaluation; SOFA, sequential organ failure assessment; HDL, high-density lipoprotein; LDL, low-density lipoprotein; APO, A-I apolipoprotein A-I; CRP, C-reactive protein.

### Lipid profile and mortality

**Figure 1:**
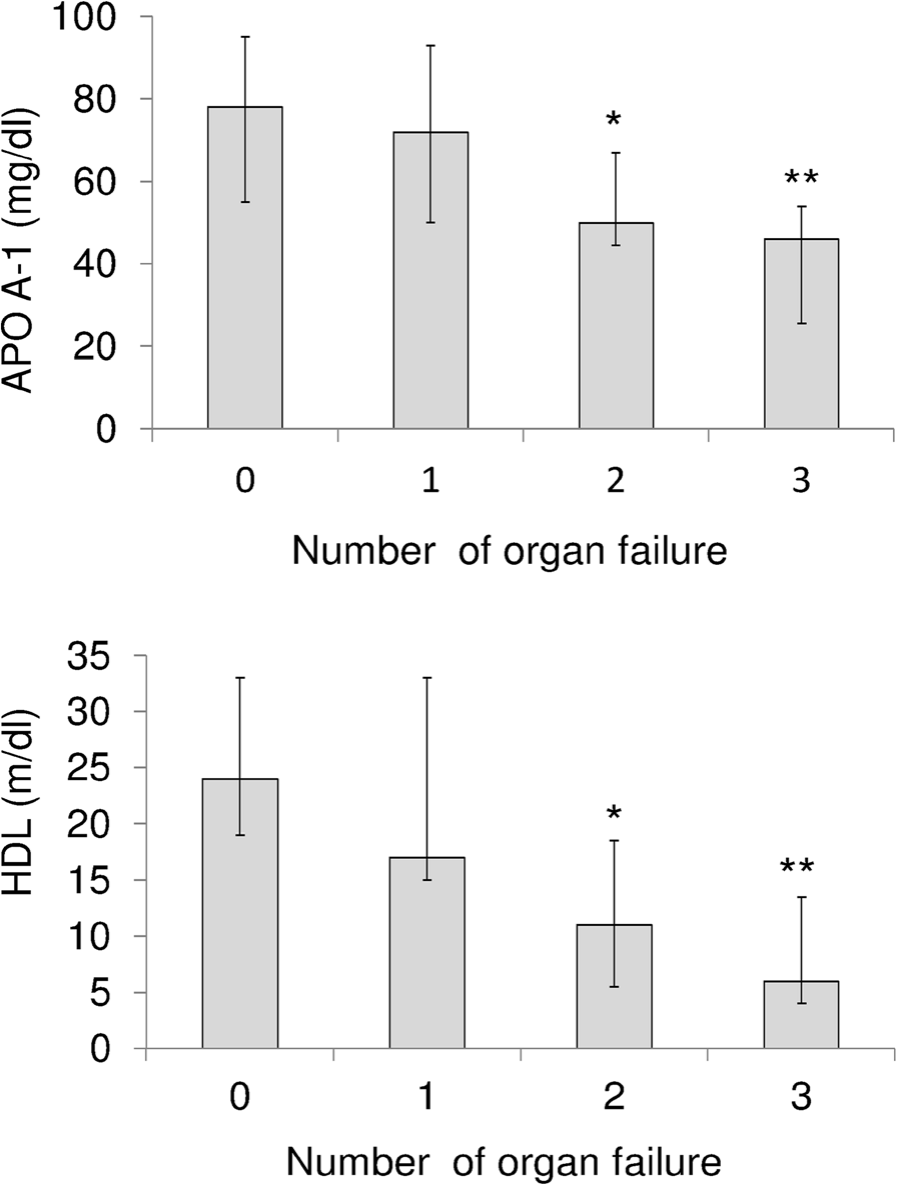
**Association among apolipoprotein A-I (APO A-1), high-density lipoprotein (HDL) and organ failure.** The levels of apolipoprotein A-I and HDL decrease progressively and significantly with the number of organ failures. *P <0.05 compared to the group with no organ system failure; **P <0.001 compared to the group with no organ system failure (Kruskall-Wallis test followed by Dunn’s post hoc test). Results are expressed as median, with error bars representing the IQR.

The levels of HDL, and APO-A-I were significantly higher in those who survived (Table 1), while the levels of TNF-α and IL-6 were higher in those who died. Although weak, HDL and APO A-I were inversely correlated with Ranson, APACHE II, SOFA and modified Marshall scores (Table 2). Among these, HDL had the strongest negative correlation with the modified Marshall score. Inflammatory cytokines were also negatively correlated with HDL, and APO A-I. Of these, HDL had the strongest inverse relationship with serum IL-6 levels. The concentrations of APO A-I and HDL decreased progressively and significantly with the number of organ failures as defined by the modified Marshall score (Figure 1). The discriminating power of APO A-I and HDL to predict 28-day mortality was tested using the AUROC. The AUROC for APO A-I and HDL to predict 28-day mortality was 0.869 ± 0.047 (mean ± standard error of the mean (SEM)), 95% CI 0.764 to 0.940, and 0.791 ± 0.059, 95% CI 0.673 to 0.881, respectively.

**Table 2:** **Correlation between serum lipid, disease severity scores, and serum inflammatory cytokines**

	**Statistics**	**Ranson score**	**APACHE II score**	**SOFA score**	**Modified Marshall score**	**TNF-α**	**IL-6**
Cholesterol	*R*	−0.198	−0.321	−0.367	−0.349	−0.335	−0.196
	*P*	0.115	0.010	0.003	0.004	0.025	0.192
HDL	*R*	−0.376	−0.483	−0.613	−0.669	−0.450	−0.540
	*P*	0.002	<0.001	<0.001	<0.001	0.001	<0.001
LDL	*R*	−0.165	−0.371	−0.335	−0.423	−0.285	−0.356
	*P*	0.196	0.003	0.008	0.001	0.057	0.018
Triglyceride	*R*	0.130	0.071	0.094	0.246	−0.012	0.185
	*P*	0.301	0.579	0.463	0.048	0.937	0.219
APO A-I	*R*	−0.378	−0.467	−0.569	−0.597	−0.467	−0.471
	*P*	0.002	<0.001	<0.001	<0.001	0.001	0.001

HDL, high-density lipoprotein; LDL, low-density lipoprotein; APO A-I, apolipoprotein A-I; APACHE, acute physiology and chronic health evaluation; SOFA, sequential organ failure assessment.

### Lipid profile and organ failure

Table 3 lists the demographic data, clinical characteristics and lipid profiles in patient subgroups stratified by the presence of OF. The levels of total cholesterol, HDL, LDL and APO A-I were significantly lower in patients with OF, while the levels of TNF-α, IL-6 and CRP were lower in those without OF. When persistency of organ failure was not taken into account, there was no difference in mortality rates between patient with OF and those without OF (Table 3).

**Table 3:** **Patients’ demographic data and clinical characteristics stratified by early organ failure**

	**Organ failure**	**No organ failure**	***P*** **-value**
	**(n = 55)**	**(n = 11)**	
Age, years	59 (45 to 76)	69 (45 to 92)	0.429
Gender, M/F, n	34/21	4/7	0.182
BMI, kg/m^2^	25.7 ± 3.8	26.2 ± 4.4	0.748
Body weight, kg	68.3 ± 12.2	65.7 ± 10.9	0.545
Etiology, alcohol/biliary/ERCP/others	21/30/1/3	4/7/0/0	0.815
Leukocytes, ×10^9^/l	13.1 ± 6.6	9.1 ± 4.6	0.058
Hemoglobin, g/dl	10.4 ± 2.3	10.3 ± 1.5	0.936
Platelets, ×10^9^/l	138 (72 to 229)	139.5 (58.5 to 247.3)	0.935
BUN, mg/dl	23 (12.5 to 41.5)	12 (7 to 22)	0.023
Serum Creatinine, mg/dl	1.5 (0.9 to 3.2)	0.7 (0.6 to 0.9)	<0.001
Bilirubin, mg/dl	1.6 (1.1 to 5.4)	1.5 (1 to 4)	0.642
INR	1.2 (1.1 to 1.5)	1.05 (1 to 1.13)	0.002
Ransons score	5.5 (4 to 7)	4 (3 to 4)	0.001
APACHE II score	18 (11 to 25.5)	14 (10 to 15)	0.028
SOFA score	6 (4.3 to 9.8)	3 (2 to 5)	0.005
Modified Marshall score	5 (3 to 7.25)	1 (0 to 1)	<0.001
Infected necrosis, n	18 (32.7%)	0 (0%)	0.028
Cholesterol, mg/dl	117 (83 to 142)	151 (109 to 172)	0.046
Triglyceride, mg/dl	148 (105 to 285)	132 (92 to 167)	0.242
HDL, mg/dl	15 (6 to 22)	24 (19 to 33)	0.010
LDL, mg/dl	56 (37 to 75)	82 (65 to 100)	0.005
APO A-I, mg/dl	54 (41 to 74)	78 (55 to 96)	0.020
IL-6, pg/ml	129 (54 to 251.5)	29 (10.4 to 101.5)	0.005
TNF-α, pg/ml	10.8 (4.7 to 23)	2.9 (1.1 to 3.7)	0.001
CRP, mg/l	190.6 ± 108.9	111.0 ± 90.4	0.027
Hospital mortality	13 (23.6%)	0 (0%)	0.104
ICU mortality	9 (16.4%)	0 (0%)	0.337
28-day mortality	11 (20%)	0 (0%)	0.188
ICU stay, days	8 (5 to 14.5)	5 (5 to 11)	0.510

Results are presented as mean ± SD, or median (IQR) unless otherwise stated. M, male; F, female; BMI, body mass index; ERCP, endoscopic retrograde cholangiopancreagraphy; BUN, blood urea nitrogen; INR, international normalized ratio; APACHE, acute physiology and chronic health evaluation; SOFA, sequential organ failure assessment; HDL, high-density lipoprotein; LDL, low-density lipoprotein; APO A-I, apolipoprotein A-I; CRP C-reactive protein.

### HDL and APO A-I as markers to predict persistent organ failure

To further demonstrate the association among lipid profile, persistent OF and mortality, those patients with OF were sub-grouped into transient OF and persistent OF. Table 4 shows the clinical characteristics and outcomes in patient subgroups stratified by persistency of OF. The levels of total cholesterol, HDL, LDL and APO A-I were significantly lower in patients with persistent OF, while the levels of TNF-α, and IL-6 were higher in those with persistent OF. The mortality rates were significantly higher in patients with persistent OF, while ICU stay was shorter in patients with transient OF. The discriminating power of APO A-I and HDL to predict persistent OF was also tested using AUROC. When all patients with predicted SAP were analyzed, the AUROC for APO A-I and HDL to predict persistent OF was 0.898 ± 0.043 (mean ± SEM), 95% CI 0.813 to 0.983, and 0.912 ± 0.036, 95% CI: 0.842 to 0.982, respectively. There was no significant difference in discriminating power between HDL and APO A-I (*P* = 0.587). When those patients with OF were analyzed, the AUROC for APO A-I and HDL to predict persistent OF was 0.895 ± 0.045 (mean ± SEM), 95% CI 0.808 to 0.983, and 0.904 ± 0.037, 95% CI 0.832 to 0.976 respectively (*P* = 0.838). These results indicate that both HDL and APO A-I are good markers in this clinical setting. Meanwhile, we also evaluated the discriminating ability of CRP because it has been used as a biomarker to predict severity [1]. The area under ROC curve for CRP obtained by analyzing all patients or patients with OF was 0.603 ± 0.061, and 0.568 ± 0.076, respectively. Pair-wise comparisons of AUROC showed that both HDL and APO A-I gave significantly higher AUROC and thus, better predictive accuracy than CRP when analyzing all patients (*P* <0.001 relative to both HDL and APO A-I) or patients with OF (*P* <0.001 relative to both HDL and APO A-I). The best cutoff points of HDL and APO A-I to predict persistent OF remained the same when analyzing all patients or patients with OF. Table 5 shows the predictive values of the chosen cutoff points (16.5 mg/dl for HDL, 64 mg/dl for APO A-I), which gave the best Youden index, for prediction of persistent OF.

**Table 4:** **Patients’ demographic data and clinical characteristics stratified by persistent organ failure**

	**Organ failure (transient)**	**Organ failure (persistent)**	***P*** **-value**
	**(n = 20)**	**(n = 35)**	
Age, years	70.5 (46.5 to 80.3)	54 (44 to 71)	0.159
Gender, M/F, n	10/10	24/11	0.173
BMI, kg/m^2^	26.5 ± 4.3	25.3 ± 3.6	0.300
Body weight, kg	66.4 ± 12.7	69.3 ± 12.3	0.415
Etiology, alcohol/biliary/ERCP/others	4/15/0/1	17/15/1/2	0.126
Leukocytes, ×10^9^/l	12.9 ± 5.6	13.20 ± 7.3	0.888
Hemoglobin, g/dl	10.5 ± 2.6	10.30 ± 2.2	0.718
Platelets, ×10^9^/l	157.5 (104.5 to 240)	112 (49 to 229)	0.178
BUN, mg/dl	16.5 (7.5 to 26)	26.5 (14 to 50.5)	0.011
Serum Creatinine, mg/dl	0.9 (0.8 to 1.3)	2.6 (1.1 to 3.8)	0.003
Bilirubin, mg/dl	1.5 (0.8 to 1.9)	2.6 (1.2 to 8.0)	0.081
INR	1.2 (1.1 to 1.4)	1.3 (1.2 to 1.6)	0.044
Ranson score	5 (4 to 5.5)	6 (5 to 7)	0.009
APACHE II score	12 (10 to 17.8)	22 (15 to 28)	<0.001
SOFA score	4.5 (2 to 6)	8 (6 to 12.3)	<0.001
Modified Marshall score	3 (2 to 3)	6.5 (4.8 to 10)	<0.001
Number of organ failures	1 (1 to 1.8)	2 (2 to 3)	<0.001
Respiratory failure	17 (85%)	33 (94.3%)	0.342
Renal failure	6 (30%)	26 (4.3%)	0.001
Cardiovascular failure	3 (15%)	21 (60%)	0.002
Infected necrosis	0 (0%)	17 (51.4%)	<0.001
Cholesterol, mg/dl	131.5 (117 to 170)	94 (83 to 135)	0.017
Triglyceride, mg/dl	144 (116 to 242)	153 (103 to 290)	0.868
HDL, mg/dl	30 (17 to 36)	8 (5 to 15)	<0.001
LDL, mg/dl	66 (56 to 74)	42 (30 to 77)	0.029
APO A-I, mg/dl	81 (69 to 100)	48 (31 to 54)	<0.001
IL-6, pg/ml	63.5 (43 to 136.5)	211 (104 to 552)	0.005
TNF-α pg/ml	6.3 (2.9 to 15)	12 (6.1 to 25)	0.048
CRP, mg/l	195.0 ± 91.4	188.3 ± 118.3	0.833
Hospital mortality	0 (0%)	13 (35.3%)	0.002
ICU mortality	0 (0%)	9 (25.7%)	0.019
28-day mortality	0 (0%)	11 (31.4%)	0.004
ICU stay, days	5 (3 to 9)	11 (5.8 to 17.3)	0.009

Results are presented as mean ± SD, or median (IQR) unless otherwise stated. M male; F female; BMI body mass index; ERCP endoscopic retrograde cholangiopancreagraphy; BUN blood urea nitrogen; INR international normalized ratio; APACHE acute physiology and chronic health evaluation; SOFA sequential organ failure assessment; HDL high-density lipoprotein; LDL low-density lipoprotein; APO A-I apolipoprotein A-I; TNF tumor necrosis factor; CRP C-reactive protein.

**Table 5:** **HDL and APO A-I for prediction of persistent organ failure**

	**Sensitivity**	**Specificity**	**PPV**	**NPV**	**Accuracy**
**All patients**					
HDL <16.5 mg/dl	0.886	0.839	0.861	0.867	0.864
APO A-I <64 mg/dl	0.943	0.806	0.846	0.926	0.879
**Patients with organ failure**					
HDL <16.5 mg/dl	0.886	0.850	0.912	0.810	0.873
APO A-I <64 mg/dl	0.943	0.850	0.917	0.895	0.909

HDL, high-density lipoprotein; APO A-I, apolipoprotein A-I; PPV, positive predictive value; NPV, negative predictive value.

We also evaluated the performance of different scores. We found that there is no difference in discriminating power of predicting persistent OF among HDL, APO A-I and different scores (Additional file 1: Table S1).

## Discussion

The major findings of this study are as follows: (1) low levels of APO A-I and HDL are associated with high levels of inflammatory cytokines, persistent OF, infected necrosis and hospital mortality in patients with predicted SAP admitted to ICU; (2) APO A-I and HDL can serve as biomarkers to differentiate persistent OF from transient OF in this clinical setting.

In acute patients with pancreatitis, inflammatory reaction initially takes place within the pancreas. Subsequently, inflammatory cytokines induce hepatic synthesis of acute-phase proteins and mediate distant organ failure in the setting of SAP [25]. Despite improvements in critical care, the mortality rate of SAP remains high and is closely associated with the development of persistent OF and subsequent sepsis [26]. Gut barrier failure with ensuing endotoxin translocation has been proposed as a major contributor to the development of OF in SAP [26]. Increased intestinal permeability occurs early after onset of acute pancreatitis and closely correlates with disease severity, levels of endotoxemia, and release of inflammatory cytokines, which initiate and propagate the deleterious consequences of severe inflammation, such as organ failure and mortality [26,27]. Recently, the capability of lipoprotein to bind and neutralize endotoxin has drawn more and more attention. When endotoxin was added to normal human whole blood *in vitro*, the binding of endotoxin to lipoproteins displays high specificity, with HDL binding the majority of endotoxin (60%) [28]. In this regard, serum lipoprotein may represent an integral part of innate immunity against organ injury in SAP.

In addition to the ability to neutralize endotoxin, HDL has also been shown to have antioxidant and anti-inflammatory properties [29]. In this regard, free radicals and oxidative stress have been implicated in the pathogenesis of acute pancreatitis and have been shown to correlate with the severity of pancreatitis [30,31]. Consistent with previous reports on critically ill patients [32-34], we showed that the levels of HDL and APO A-I at admission to ICU were significantly lower in non-survivors. We also demonstrated that the levels HDL and APO A-I were significantly lower in patients with persistent OF, while the levels of TNF-α, and IL-6 were lower in those with transient OF. Although hypolipidemia is a well-recognized phenomenon in critical illness [32,33], the mechanisms are incompletely understood and probably multi-factorial. Both decreased synthesis and enhanced catabolism may be involved. It has been shown that TNF-α and IL-6 can dose-dependently inhibit synthesis of apolipoprotein in hepatic cell lines [15]. On the other hand, over-expression of secretory phospholipase A2, an acute-phase protein, can induce rapid catabolism of HDL and APO A-I [35]. Taken together, high levels of inflammatory cytokines in patients with SAP may not only impair the biosynthesis of APO A-I and HDL but also facilitate the degradation of lipoprotein, rendering these patients even more susceptible to harmful consequences of inflammation. Additionally, increased levels of endotoxin in SAP may overwhelm the already impaired neutralization ability provided by low levels of HDL and APO A-I and subsequently become even more unopposed, thus perpetuating the overproduction of inflammatory cytokines. A vicious cycle ensues and leads to further failure of multiple organ functions. Agreeing with this hypothesis, we showed that serum levels of APO A-I were inversely correlated with systemic inflammatory states, which was indicated by serum levels of TNF-α and IL-6 (Table 2). We also demonstrated that the concentrations of APO A-I and HDL decreased progressively as the number of organ failures increased (Figure 1).

Another novel finding of our study is that APO A-I and HDL can differentiate persistent OF from transient OF. In this regard, the existing scoring systems have shown moderate accuracy in predicting persistent OF [36]. However, these scoring systems are cumbersome to use and appeared to have reached their maximal efficacy in this clinical setting. To improve the capability of predicting persistent OF, clinicians need to develop new approaches. In addition to recognized scoring systems, recent attention has also focused on simple biomarkers that could give predictive information [37,38], such as blood urea nitrogen (BUN) and creatinine. Despite these encouraging results, there are drawbacks in currently available data that these markers are not necessarily patho-physiologically linked to the development of the OF and therefore, elevation of these markers simply reflects ongoing OF. Theoretically, patho-physiologically relevant biomarkers may perform better in this clinical setting. In this regard, blood lipoprotein may provide important prognostic information and help risk stratification.

There are limitations of our study. First of all, we studied these markers in patients admitted to ICU, therefore, our results may not be generalizable in those patients with less severe illness. Second, the number of patients studied was small. Further studies with larger cohorts are needed to validate our results.

## Conclusions

The concentrations of HDL and APO A-I are inversely related to disease severity in patients with predicted SAP admitted to ICU. Serum levels of HDL and APO A-I at admission to ICU can predict persistent OF in this clinical setting. Whether lipid-based treatment can play a therapeutic role in the management of SAP deserves further investigation.

## Key messages

- Low levels of APO A-I and HDL are associated with high levels of inflammatory cytokines, persistent OF, infected necrosis and hospital mortality in patients with predicted SAP admitted to ICU
- APO A-I and HDL can serve as biomarkers to differentiate persistent OF from transient OF in this clinical setting

## Additional file

Additional file 1: Table S1. Pair-wise comparisons of area under the receiver operating characteristic curve (AUROC) for high-density lipoprotein (HDL), apolipoprotein A-I (APO A-I) and scores to predict persistent organ failure.

## Abbreviations

APACHE: acute physiology age, and chronic health evaluation
APO A-I: apolipoprotein A-I
AUROC: area under a receiver operating characteristic curve
BMI: body mass index
BUN: blood urea nitrogen
CRP: C-reactive protein
CT: computed tomography
ERCP: endoscopic retrograde cholangiopancreagraphy
HDL: high-density lipoprotein
IL-6: interleukin-6
INR: international normalized ratio
LDL: low-density lipoprotein
NPV: negative predictive value
OF: organ failure
PPV: positive predictive value
ROC: receiver operating characteristic curve
SAP: severe acute pancreatitis
SOFA: sequential organ failure assessment
TG: triglyceride
TNF: tumor necrosis factor
