# Supplementary material for: Serum levels of apolipoprotein A-I and high-density lipoprotein can predict organ failure in acute pancreatitis
Source: Crit Care. 2015 Mar 17;19(1):88. doi: 10.1186/s13054-015-0832-x (PMC4363356; doi:10.1186/s13054-015-0832-x)
Supplement: Additional file 1: Table S1. — Pair-wise comparisons of area under the receiver operating characteristic curve (AUROC) for high-density lipoprotein (HDL), apolipoprotein A-I (APO A-I) and scores to predict persistent organ failure. [file 13054_2015_832_MOESM1_ESM.pdf]

Table S1 Pair-wise comparisons of AUROC for HDL, APO A-I and scores to predict persistent organ failure

Parameter	AUROC (95% CI)	SE	P-value[a]	P-value[b]
HDL	0.912 (0.842-0.982)	0.036	-	0.671
APO A -I	0.898 (0.813-0.983)	0.043	0.671	-
Ranson’s score	0.798 (0.674-0.891)	0.058	0.067	0.125
APACH II score	0.813 (0.691-0.902)	0.057	0.098	0.188
SOFA score	0.868 (0.755-0.941)	0.046	0.369	0.595
Modified Marshall score	0.934 (0.838-0.982)	0.030	0.652	0.499

[a]P values are given relative to the HDL

[b]P values are given relative to the APO A-I

Abbreviation: HDL, high-density lipoprotein; APO A-I, apolipoprotein I; APACHE, Acute Physiology, Age, and Chronic Health Evaluation; SOFA, sequential organ failure assessment; AUROC, area under a receiver operating characteristic; SE, standard error
